# Layperson-Friendly AI Translation of Medical Documents to Improve Doctor–Patient Communication: Protocols for the AI-INFOCARE and AI-MEDTALK Randomized Controlled Trials

**DOI:** 10.2196/77204

**Published:** 2025-11-21

**Authors:** Elida Hasani, Sven Richter, Tareq Adnan Juratli, Clara Helene Buszello, Markus Georg Prem, Sophia Willkommen, Sahr Sandi-Gahun, Ilker Yasin Eyüpoglu, Witold Henryk Polanski

**Affiliations:** 1 Department of Neurosurgery Medical faculty Carl Gustav Carus of the TU Dresden University Hospital Carl Gustav Carus Dresden Germany

**Keywords:** artificial intelligence, autonomy support, doctor–patient communication, health literacy, patient education, randomized controlled trial

## Abstract

**Background:**

Many patients struggle to understand referral letters and discharge summaries; low health literacy is prevalent, and short consultations limit explanations. Large language models (LLMs) can translate clinical jargon into layperson language, but their impact on doctor–patient communication in real care remains untested.

**Objective:**

This study aims to determine whether providing an artificial intelligence (AI)–generated, medically validated layperson-language translation of key medical documents before a consultation improves the quality of doctor–patient communication.

**Methods:**

We plan to conduct two single-center, parallel-group randomized controlled trials in neurosurgery: AI-INFOCARE (outpatients prior to treatment discussions) and AI-MEDTALK (inpatients at surgical discharge). Adults (aged 18 years or older, German-speaking, and able to consent) are randomized 1:1 to receive either (1) an AI-generated layperson-friendly summary of their referral or discharge document in addition to usual care or (2) usual care only. Summaries are produced with a Claude-based system (Simply Onno GmbH) and undergo human verification against the source document using a predefined checklist. The primary outcome is patient-rated interaction quality (Fragebogen zur Arzt-Patient-Interaktion/Questionnaire on the Quality of Physician–Patient Interaction) immediately postconsultation. Secondary outcomes are perceived autonomy support (brief Health Care Climate Questionnaire), self-rated understanding (5-point item), physician-rated encounter difficulty (Difficult Doctor–Patient Relationship Questionnaire, 10-item), and consultation length (minutes). Randomization uses a computer-generated allocation list with concealed, sequential assignment at the point of inclusion; recruiting clinicians have no access to the sequence, and analysts are blinded to group codes. Fidelity is assured by standard operating procedures, rater training, and periodic dual review with interrater agreement estimates. Safety monitoring defines information-related adverse events (eg, distress requiring unplanned clinical support or clinically relevant inaccuracies) and includes an independent safety overseer. Ethics approvals were obtained in February 2025 from the ethics committee of Technische Universität Dresden, and both trials are registered in the German Clinical Trials Register.

**Results:**

Each trial targets 300 participants (150 per arm), providing greater than 80% power for an effect size *d*≈0.4 on the primary outcome (α=0.05). Outcomes are assessed immediately after the index consultation, with no additional follow-up planned in this protocol. We hypothesize that providing layperson-friendly summaries will significantly improve patients’ understanding and satisfaction with information, foster a more autonomy-supportive communication climate, and reduce physicians’ perceived difficulty in the encounter without unduly prolonging consultation time. Neither study received external funding. Both trials are currently in the recruitment phase, with patient enrollment scheduled to begin in May 2025 and expected to conclude by February 2026. Results are anticipated to be published in summer 2026.

**Conclusions:**

These pragmatic randomized controlled trials test a scalable AI intervention to strengthen understanding and interaction quality without adding clinician burden. If effective, AI-assisted layperson summaries could be integrated into routine workflows to advance health literacy and patient-centered care.

**Trial Registration:**

Deutsches Register Klinischer Studien DRKS00036810; https://www.drks.de/search/de/trial/DRKS00036810 and Deutsches Register Klinischer Studien DRKS00036814; https://drks.de/search/de/trial/DRKS00036814

**International Registered Report Identifier (IRRID):**

PRR1-10.2196/77204

## Introduction

Quality communication between physicians and patients is a cornerstone of effective health care. It is well documented that better doctor–patient communication improves patient understanding, satisfaction, adherence to treatment, and even clinical outcomes [1]. Conversely, communication breakdowns can undermine the therapeutic relationship and health results. Effective communication serves multiple purposes: building a good interpersonal relationship, exchanging information, and engaging patients in decision-making [2]. Achieving these goals requires that patients comprehend the information conveyed.

However, many patients struggle to understand complex medical terminology used in both verbal consultations and written documents such as specialist referral letters, procedure reports, or hospital discharge summaries. In Germany and internationally, limited health literacy is a pervasive challenge. A recent national survey found that 58.8% of German adults have inadequate health literacy, reporting difficulty understanding and using health information [3]. Such low health literacy is associated with poorer communication, lower patient engagement, and worse health outcomes [3]. Schaeffer et al [3] noted that improving the understandability of patient information is urgently needed to address this gap.

Doctor–patient communication can be conceptualized through the dual lenses of health literacy and shared decision-making (SDM). According to Leventhal’s Common-Sense Model and SDM frameworks, understandable information is a prerequisite for autonomy support and meaningful participation in care. Our outcomes map onto these constructs: the Fragebogen zur Arzt-Patient-Interaktion (FAPI)/Questionnaire on the Quality of Physician–Patient Interaction (QQPPI) captures interaction quality, the brief Health Care Climate Questionnaire (HCCQ) reflects autonomy support, and self-rated understanding measures the informational pathway. By improving document comprehensibility before an encounter, the intervention directly targets these theoretical pathways expected to enhance both informational and relational aspects of communication.

Compounding the issue, physicians often face severe time constraints during appointments. Germany is among the countries with the shortest primary care visit lengths, historically averaging only about 7 to 10 minutes per patient [4]. This limited time makes it difficult to explain diagnoses and treatments in depth, especially if patients are encountering complex information for the first time. Studies indicate that physicians feel pressure to cover medical content quickly, which can lead to inadequate explanations and patients leaving with unanswered questions [4]. Time pressure has been linked to decreased patient recall and satisfaction [1]. Many patients turn to external sources, such as the internet, for clarification, which may yield unreliable information (“Dr Google”). In the worst cases, misunderstanding or lack of information can cause patient anxiety, unnecessary repeat consultations, or even nonadherence and premature therapy discontinuation [5,6].

Written medical documents are a key part of doctor–patient communication but are often written primarily for other health care professionals, not laypersons. For example, a hospital discharge letter typically contains medical jargon, abbreviations, and complex narrative that an average patient finds hard to decipher. This information gap represents a missed opportunity: if patients could better understand their medical records, they would be more empowered during follow-up visits with their doctors. Past efforts to improve this have included manually creating simplified, patient-friendly discharge letters. A preliminary trial in Austria demonstrated that providing patients (or proxies) with a simplified discharge summary significantly improved their acceptance of and satisfaction with the information compared to the standard technical letter [7]. Participants valued the clearer structure, avoidance of abbreviations, and layperson language, indicating that patient-friendly documents enhance communication.

Recent advances in artificial intelligence (AI) now enable automated translation of complex text into simpler language. Large language models (LLMs) such as GPT-4, Gemini, Llama, and Claude can parse medical text and generate explanations in more digestible terms. Early studies suggest that AI-generated summaries can dramatically improve readability. Zaretsky et al [8] showed that an LLM-transformed discharge summary achieved a mean reading level of sixth grade versus eleventh grade for the original and an understandability score of 81% versus 13%, without losing accuracy (although some errors occurred). Similarly, a Korean study found that ChatGPT could produce patient-friendly discharge notes that were easier to read, highlighting AI’s potential in multilingual settings. These findings underscore that AI can serve as a powerful tool to bridge the language gap in medical communication, provided its outputs are properly validated for accuracy and completeness [8]. Compared with manual simplification approaches, which improve comprehension but are resource-intensive and difficult to scale, LLM-based systems offer immediate generation at low marginal cost. However, AI introduces distinct risks, such as hallucinations or omissions, which necessitate systematic human validation. Our protocol therefore emphasizes a fidelity process with predefined checklists, dual review of samples, and correction logging to balance scalability with safety.

To our knowledge, no completed randomized controlled trials (RCTs) have yet evaluated AI-generated, medically validated layperson translations of medical documents delivered before real-world consultations. However, related trials in digital health literacy and patient-oriented discharge summaries are ongoing or recently registered, underscoring the timeliness and originality of this study. For example, in Germany, study DRKS00035739 (“PALLADS”) is currently recruiting and testing whether LLM-assisted simplification of discharge summaries improves patient activation; similarly, NCT06859216 examines AI-generated plain-language versions of ophthalmology notes. These confirm that although related trials are underway, none to date have completed RCTs of AI-generated, medically validated layperson translations of referral or discharge documents delivered before real-world consultations. The AI-INFOCARE and AI-MEDTALK trials have been conceived to fill this gap. These two parallel RCTs will test the central hypothesis that giving patients an easy-to-understand version of their medical documents before they speak with their doctor will lead to more effective and satisfying communication during the consultation. Specifically, we expect that patients with the AI-assisted information will feel more informed and autonomous, ask better questions, and overall experience a better-quality interaction with their physician, whereas doctors may perceive less difficulty in explaining and managing the visit.

Both trials are prospective, two-arm randomized controlled studies conducted at a large academic medical center in Germany (the Neurosurgery Clinic at Universitätsklinikum Carl Gustav Carus, Dresden). Importantly, they address two complementary patient populations and clinical contexts: AI-INFOCARE focuses on outpatients about to undergo treatment (improving understanding before a treatment discussion), whereas AI-MEDTALK focuses on inpatients at discharge after surgery (improving understanding of what happened and next steps). By covering both “front-end” and “back-end” clinical scenarios, the trials together aim to generate broadly applicable evidence on this AI intervention.

If successful, these studies will produce evidence for a scalable digital health intervention that could be implemented across various settings to improve patient comprehension and engagement. Enhancing communication in this way aligns with public health goals of improving health literacy and patient-centered care. Ultimately, clearer initial understanding can reduce follow-up questions, prevent misinformed decisions, and strengthen the therapeutic alliance [1]. We also acknowledge potential challenges: for instance, doctors must trust and verify AI outputs (to avoid any misinformation), and workflows must integrate generating and sharing summaries without causing delays. The Discussion section will address these considerations.

In summary, the trials will test an innovative strategy to achieve the age-old goal of “understanding instead of uncertainty” in health care communication. This manuscript presents the detailed protocol following SPIRIT guidelines, including rationale, methods, and planned analyses for AI-INFOCARE and AI-MEDTALK.

## Methods

### Ethical Considerations

This study was approved by the ethics committee of Technische Universität Dresden. The approval numbers are AI-INFOCARE: BOff(Mono)-EK-89022025 and AI-MEDTALK, BOff(Mono) -EK-91022025. The study protocol was prepared in accordance with SPIRIT (Standard Protocol Items: Recommendations for Interventional Trials) guidelines for clinical trial protocols and approved by the Technische Universität Dresden ethics committee in February 2025. Both trials are registered in the German Clinical Trials Register (DRKS00036810 and DRKS00036814). All participants will provide informed consent. We take care to ensure the intervention does not negatively impact care: the AI summaries are provided in addition to usual explanations, not replacing physician communication. Physicians are instructed to verify and correct any misunderstanding. We also ensure confidentiality of patient data; the AI processing is conducted on a General Data Protection Regulation/Datenschutz-Grundverordnung–compliant server, and no data are shared beyond the study team. Potential risks to participants are minimal—there is a slight risk that a patient might misinterpret an AI summary if it contained a minor error, but our human verification mitigates this. We also considered the psychological impact: for instance, reading one’s medical details in plain language could be distressing if the news is bad (eg, diagnosis of a serious illness). In standard care, patients might not fully grasp the seriousness from the medical jargon, whereas the plain summary could make it clear. This is ethically complex, but we believe transparency is ultimately better for informed decision-making. Doctors will be available to discuss any concerns raised by reading the summary. Indeed, this may prompt important discussions that otherwise would not have occurred.

We define information-related adverse events (AEs) as (1) patient distress from the summary requiring unplanned clinical support during or after the visit, (2) clinically relevant inaccuracies identified in the summary, or (3) formal complaints linked to the intervention. All AEs are recorded on standardized forms and reviewed quarterly by an independent safety overseer; serious AEs are reported within 72 hours.

No monetary compensation is provided, but we find that patients are generally eager to participate in order to possibly receive a clearer explanation.

Lastly, we disclose that WHP is affiliated with Simply Onno GmbH, which developed the AI-based translation. This relationship is noted as a potential conflict of interest. To ensure unbiased conduct, outcome assessments rely on participant responses, and data analysis will be overseen by the academic investigators. There is no external funding for this study; it is supported by internal resources of the hospital and Simply Onno GmbH. The absence of external funding is declared to maintain transparency.

### Study Design and Setting

Both AI-INFOCARE and AI-MEDTALK are designed as prospective, parallel-group RCTs. The trials share a common overall design, with nearly identical interventions and outcomes, but target different patient populations and phases of care (outpatient pretreatment vs inpatient discharge). The study setting is the Department of Neurosurgery at University Hospital Carl Gustav Carus, Technische Universität Dresden, Germany, which includes outpatient clinics and inpatient wards. The trials are investigator-initiated collaborations between the hospital and Simply Onno GmbH (Berlin; [9]), a health AI startup.

**Figure 1:**
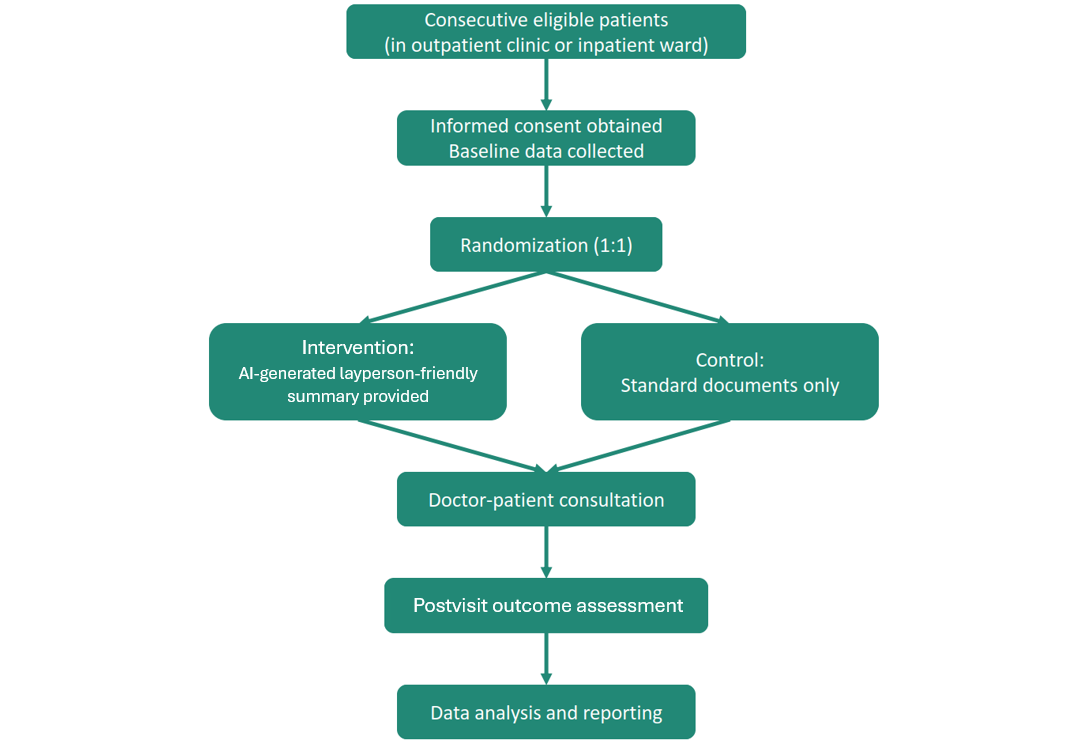
Flow diagram of the trial design for AI-INFOCARE and AI-MEDTALK. Both trials follow the same procedure: eligible patients are approached and consented, then randomized 1:1 to receive either the artificial intelligence (AI)–generated layperson summary of their medical document (intervention) or standard care with no summary (control) before their doctor consultation. The consultation proceeds as usual, and afterward outcomes are assessed through questionnaires and data collection. Finally, data will be analyzed comparing the two groups.

The overall structure of the trials is summarized in Figure 1. Each trial will enroll participants over a 6-month recruitment period and follow a simple two-arm randomized design (intervention vs control). There is no blinding (open-label) and no crossover between arms. Outcome assessments are conducted at a single postintervention time point (immediately after the index doctor–patient consultation during which the intervention could have an effect). There are no long-term follow-ups in this protocol, as the primary focus is on the immediate communication outcomes of the consultation.

### Participants and Recruitment

Each trial will recruit approximately 300 patients (150 per arm), for a total of 600 participants across both trials. Participants will be recruited consecutively from the patient population served by the neurosurgery clinic. AI-INFOCARE will enroll ambulatory patients before a planned treatment, and AI-MEDTALK will enroll hospitalized patients at discharge after surgery.

#### AI-INFOCARE (Outpatient Cohort)

Eligible patients are adults scheduled for a neurosurgery outpatient appointment in preparation for a procedure or treatment discussion. Examples include patients referred for elective neurosurgical procedures (e.g. tumor resection, spine surgery) who have prior medical reports (such as referral letters, imaging reports) that will be discussed during their consult. Enrollment will occur when the patient arrives for their pretreatment consultation or is identified shortly beforehand via appointment lists.

#### AI-MEDTALK (Inpatient Cohort)

Eligible patients are adults who have undergone a neurosurgical operation and are now being discharged from the hospital. Typically, such patients receive a discharge summary document and a briefing from the surgical team about the operation findings, postoperative care, and follow-up. Enrollment will occur on the day of discharge, prior to the discharge consultation.

#### Inclusion Criteria (Both Trials)

Eligible participants are adults aged 18 years or older and capable of providing informed consent, with sufficient German language proficiency to read the documents and complete questionnaires (the intervention and all outcome measures are in German). For AI-INFOCARE, the patient must have at least one relevant medical document available, such as a referral letter or diagnostic findings, that can be provided to them. For AI-MEDTALK, a written discharge summary or report must be prepared as per standard care, which can be used for the intervention. Participants must be willing to take part in the study and able to complete a brief survey after the consultation.

#### Exclusion Criteria (Both Trials)

Exclusion criteria include patients with severe cognitive impairment, altered mental status, or diagnosed dementia or delirium that would prevent comprehension of materials or completion of surveys. Patients who are unable to read (due to illiteracy or severe visual impairment that cannot be corrected) or unable to communicate in German are also excluded. Patients in emergency situations or acute distress where the study procedures could interfere with urgent care, such as an acute postoperative complication requiring intensive care, are not eligible. Inpatients who have had an extremely prolonged hospital stay with multiple prior discussions, which might dilute the effect of a one-time summary at discharge, are excluded. Any patient whom the treating physician deems inappropriate for the study—for example, due to extreme anxiety or other psychosocial factors, defined as inability to complete consent or questionnaires despite support or requiring acute intervention documented by staff—is also excluded. Finally, participation in another interventional study that could conflict with outcomes, or prior enrollment in the current trials (to avoid duplicate participation if a patient were first an outpatient and later an inpatient) renders a patient ineligible; in such cases, they would only be enrolled in one of the two trials.

#### Screening and Recruitment

A study team member (a trained research nurse or physician) will screen upcoming clinic appointments and inpatient lists daily to identify potential participants meeting the above criteria. Recruitment is consecutive, meaning every patient who meets criteria is approached without selection beyond eligibility, to minimize sampling bias. Patients will be provided written and oral information about the study, and written informed consent will be obtained by study personnel prior to randomization. The general nature of the intervention (receiving an AI-prepared explanation) will be explained, but participants will not be explicitly told whether their documents have been simplified by AI until after the consultation (to avoid expectation bias, although patients in the control arm will likely notice the absence of a simplified text). They are told that the study is comparing different ways of providing information.

At the time of consent, baseline data will be collected, including demographics (age, sex, education level) and clinical context (diagnosis, type of document available). No identifiable personal health data beyond what is needed for outcomes will be retained in the research dataset, and all AI processing of documents occurs on secure servers in adherence with privacy regulations (no patient-identifying information is input to the AI model). Participants are free to withdraw at any time. We anticipate high acceptability of the intervention; similar studies have reported that patients appreciate receiving additional explanations [7].

### Randomization and Blinding

Randomization was performed using a computer-generated randomization list created prior to study start. The list was produced with a validated random number generator, fully covering the planned sample size for each trial (n=300). Assignments were generated in a 1:1 ratio (intervention vs control) and exported into a password-protected file with time-stamp documentation. The randomization list was stored on a secure hospital server, accessible only to the principal investigator and the designated study statistician. Allocation concealment was maintained because recruiting staff and treating physicians had no access to the sequence; group assignment for each newly enrolled participant was revealed sequentially through a locked module at the point of inclusion. This procedure prevents prediction of future allocations while ensuring reproducibility and auditability.

Due to the nature of the intervention, blinding is not possible for participants or treating physicians. Patients in the intervention arm will receive an additional written summary that is clearly different from standard documents, and physicians will likely recognize if a patient has an easy-summary or not (eg, patients might refer to it during the consult). We considered having a blinded outcome assessor, but our outcomes are primarily self-reported by patients and the treating physician immediately after their encounter, so blinding is not applicable. The study analysts will remain blind to group labels until after data analysis (groups will be coded as A and B during statistical analysis to prevent subconscious bias). While lack of blinding may introduce some risk of bias, outcomes such as questionnaire scores are less prone to observer bias and more a reflection of the participant’s genuine experience. We will compare certain objective measures (like consultation length) to see if any Hawthorne effect (change in behavior due to being studied) is present similarly in both groups.

To mitigate expectancy and performance bias, we use several safeguards: (1) all participants receive a standardized neutral script at enrollment (“You will receive information materials that may vary in format”); (2) clinicians are instructed not to explicitly reference the AI summaries; (3) objective endpoints, such as consultation time, complement self-reported measures; (4) the analyst is blinded to group codes; and (5) sensitivity analyses adjusted for age, education, and clinician identifier will be conducted.

### Intervention: AI-Generated Layperson Summary

#### Intervention (AI-Based Translation)

Participants randomized to the intervention will receive a layperson-friendly summary of their medical documents, generated by an AI language model, in addition to the standard care documents. Specifically, for each patient, we will take the relevant medical text (eg, referral letter from the general practitioner detailing the condition and prior investigations, or the hospital discharge summary, which includes diagnosis, procedures, and follow-up recommendations) and process it through an AI system that produces an easy-to-understand version in German. The AI system, provided by Simply Onno GmbH, is built on the Claude 3.5 LLM (Anthropic), which has been configured through prompt engineering for patient-facing simplification. The model was instructed to rewrite the input document in clear, simple language targeting approximately a B1 reading level (roughly middle school reading ease). It replaces medical jargon with everyday terms (or explains them), expands abbreviations, and structures the information into a patient-oriented format (for example, using short bullet points or headings for diagnosis, treatment, and next steps).

Before being given to the patient, each AI-generated summary undergoes a quick verification by a member of the study team (typically a physician or medically trained researcher) to catch any obvious errors or unsafe omissions. This step is essential because LLMs can occasionally produce inaccuracies (“hallucinations”); any such instance will be corrected by the reviewer. In practice, the review has shown the summaries to be accurate, with occasional need to adjust a phrase. The final approved summary, typically 1-2 pages in length, is then provided to the patient before their consultation with the doctor.

To ensure fidelity, reviewers follow a 12-item checklist covering accuracy of diagnoses and procedures, completeness of follow-up instructions, preservation of risk or urgency language, and absence of contradictions or invented content. Ten percent of summaries undergo dual review, with interrater agreement reported (Cohen κ and intraclass correlation coefficient). A predefined correction log template (Multimedia Appendix 1) will be used to categorize and document any issues detected during validation. As the trial has not yet commenced, no entries are available at this stage.

For copyright and intellectual property reasons, the exact prompts cannot be disclosed. However, all prompts were version-controlled, and each version was archived with time-stamps in the study’s secure documentation system. Any changes in prompt wording or model parameters were logged and linked to the summaries generated during that phase. This ensures transparency and reproducibility of the process without requiring disclosure of proprietary content.

For outpatients (AI-INFOCARE), the summary is handed to them at the clinic check-in, so they can read it while waiting. For inpatients (AI-MEDTALK), the summary is given on the morning of discharge, a few hours before the formal discharge meeting with the physician. Patients are encouraged to read the summary and note any questions they have. They are told that this is “an explanation of your medical letter in simple language that you can keep.”

The physician conducting the consultation is aware that the patient has this summary (and in many cases, the physician will have seen it, since it mirrors the content of their own letter but in simpler form). However, physicians are instructed to conduct the consultation as they normally would, with no special training or script from the study. They will naturally address patient questions or clarify misunderstandings as needed. We will later examine whether having the summary changed the nature of questions patients ask or the flow of conversation (captured indirectly through outcomes such as consultation length and perception measures).

#### Control (Usual Care)

Participants randomized to the control arm go through usual care procedures without any additional intervention. For outpatients, this means they have whatever documents they normally would (often patients do bring their referral letter or test results, but these are in technical language, and we do not provide any translated version). They will have their consultation with the doctor using those standard materials. For inpatients, usual care means they receive the standard discharge summary at the end of the discharge discussion (or sometimes given to them to hand to their next doctor), but not ahead of time in simplified form. Essentially, the control group represents the status quo: the patient relies on the doctor’s oral explanation during the consultation and on the standard medical documents, with no separate plain-language text.

All other aspects of care are identical between groups. Both groups’ consultations take place with the same pool of physicians (neurosurgery attendings or senior residents), in the same settings. Physicians will have the original medical documents in both cases for reference. After the consultation, both groups complete the outcome questionnaires described below.

Notably, if a patient in the control group expresses strong confusion about their medical documents or requests an explanation in writing, the physician will handle it per usual care (which might involve some oral explanation or writing brief notes, but the prepared AI summary will not be provided at that point, to preserve the integrity of the comparison). However, at the end of the study, control patients will be offered the layperson-friendly summary if they desire, as an ethical consideration to avoid denying potentially helpful information.

### Outcome Measures

#### Overview

**Figure 2:**
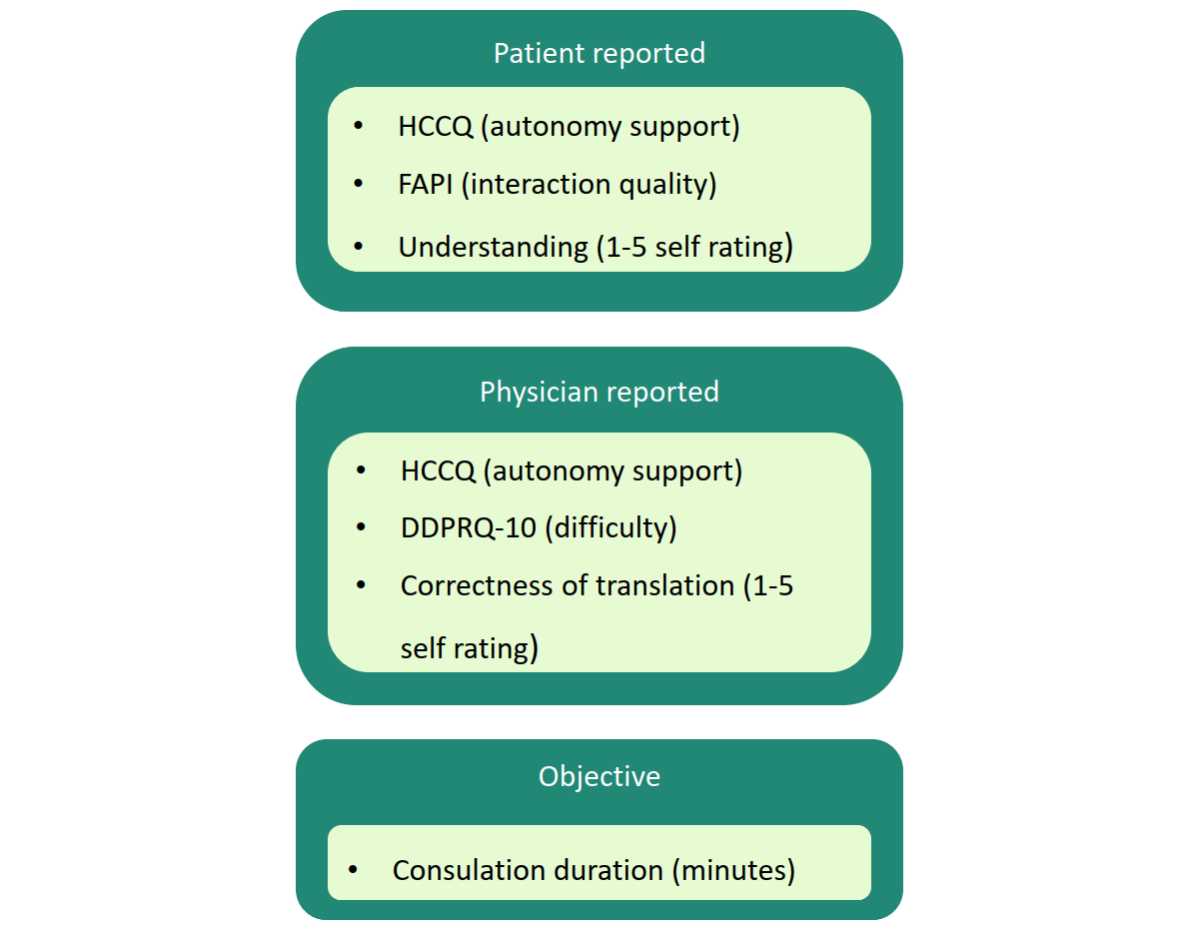
Outcome structure for communication evaluation. The trials assess patient-reported outcomes (communication quality and autonomy support through the Fragebogen zur Arzt-Patient-Interaktion [FAPI] and brief Health Care Climate Questionnaire [HCCQ], and the patient’s self-rated understanding), a physician-reported outcome (encounter difficulty through the Difficult Doctor–Patient Relationship Questionnaire [DDPRQ-10]), and an objective measure (consultation duration).

We chose outcome measures that capture multiple dimensions of doctor–patient communication and the effects of improved information comprehension. The outcomes have been validated in prior research and will be assessed for each patient (and their physician, in one measure) immediately after the consultation. Figure 2 outlines and illustrates the relationship between the outcomes and instruments, categorized as patient-reported, physician-reported, and objective measures. The specific outcome measures are described in the following sections.

#### Primary Outcome

We designate the patient’s perceived quality of the doctor–patient interaction as the primary outcome, measured by the FAPI (known in English as the Questionnaire on the Quality of Physician–Patient Interaction [QQPPI]). The FAPI is a 14-item self-report questionnaire developed and validated in Germany to gauge the quality of a specific medical consultation from the patient’s perspective. It covers aspects such as the doctor’s empathy, clarity of explanations, patient involvement in decisions, and time given. Each item is a statement (eg, “The doctor explained the treatment options in an understandable way” or “I felt the doctor took my concerns seriously”) rated on a 5-point Likert scale (“not at all” to “very much”). An overall FAPI score is calculated as the mean of item scores (after appropriate reversal of any negatively worded items), with higher score indicating better interaction quality. The FAPI has demonstrated excellent psychometric properties in previous studies, with Cronbach α around 0.95-0.97, indicating high internal consistency. It has shown convergent validity by correlating strongly with patient satisfaction and decision comfort. In these trials, the FAPI is administered in German immediately after consultation. We hypothesize that FAPI scores will be higher in the intervention group, reflecting an improved communication experience when patients have had the opportunity to understand their medical information beforehand.

#### Secondary Outcome 1: Perceived Autonomy Support From the Physician

This outcome is measured by the HCCQ. The HCCQ is a widely used instrument rooted in self-determination theory, assessing the degree to which the patient feels their doctor was autonomy-supportive versus controlling [10]. We will use the 6-item brief HCCQ (German version), with items like “My doctor encouraged me to ask questions” and “I felt understood by my physician,” rated on a 7-point Likert scale [10]. Scores are averaged, with higher scores indicating more autonomy support. The HCCQ has been validated in multiple contexts, showing strong reliability (Cronbach α approximately 0.90) [11]. This outcome captures an important facet of communication quality—whether having more knowledge through the summary makes patients feel more respected and involved in the conversation. We anticipate higher autonomy support in the intervention arm, as doctors may not need to adopt a directive stance and patients may participate more actively when they understand the information.

#### Secondary Outcome 2: Patient’s Self-Rated Understanding of the Information Discussed

This is a single-item outcome where, after the consultation, we ask the patient: “How well do you feel you understood the information and explanations given to you during this consultation?” on a 5-point Likert scale (1=not at all, 5=very well). This simple metric is commonly used in communication studies and correlates with knowledge retention. It serves as a direct subjective indicator of whether the intervention fulfilled its immediate purpose (improving understanding). We will compare the distribution of responses between groups, expecting a higher proportion of “well” or “very well” responses in the AI summary group. Even a one-point improvement on this scale would be meaningful in practice.

#### Secondary Outcome 3: Physician’s Perception of the Encounter Difficulty

This outcome is measured by the Difficult Doctor–Patient Relationship Questionnaire, 10-item (DDPRQ-10) version [12]. The DDPRQ is a survey the doctor fills out after seeing a patient, reflecting how “difficult” or frustrating the physician found the encounter. It touches on feelings like “I found this patient difficult” or “This was a frustrating visit,” rated typically on a 6-point scale [13]. We are using the 10-item short form (each item 1=not at all, 6=a lot), which has been shown to retain high reliability (Cronbach α approximately 0.93) [12]. A total score is averaged (higher score means the doctor perceived the patient as more difficult). We chose this outcome to capture the physician’s side of the communication dynamic. Often, patients who are confused, anxious, or demanding due to lack of understanding can be perceived as “difficult” by doctors [13]. We hypothesize that doctors will rate encounters in the intervention arm as less difficult on average, since patients may be more at ease and focused on pertinent questions rather than expressing confusion or dissatisfaction. Each patient’s consulting physician will complete the DDPRQ-10 immediately after the consultation, before seeing the next patient, to ensure responses reflect that specific encounter.

#### Secondary Outcome 4: Consultation Duration

We will record the length of the consultation (in minutes) from start to finish. In the outpatient setting, a research assistant will unobtrusively note the time the patient enters and leaves the doctor’s office. In the inpatient discharge setting, the time of the conversation with the doctor (often in the patient’s room or ward office) will be noted. This outcome addresses efficiency, as providing patients the summary could either increase time if patients ask more questions or discussion goes deeper, or decrease time if basic explanations need less repetition. Previous research offers mixed insights—sometimes better-informed patients make visits slightly longer due to more questions, but those questions may be more to the point, and satisfaction improves. We do not consider a modest change in time inherently negative; however, it is important to document if the intervention significantly alters physician time burdens. Our expectation is that consultation time might not differ greatly or could even decrease in the intervention group if clarification of basics is faster. This outcome is measured in a single-blind fashion, with the person timing is not influencing the interaction.

In addition to these main outcomes, we will collect exploratory data, for instance the number of questions patients ask during the consultation (not formally tallied, but physicians will provide a subjective rating of patient engagement) and any specific misunderstandings noted (physicians can report if they had to correct any wrong notions the patient had from reading the summary or otherwise). We will also debrief patients in the intervention group with a couple of questions about the summary itself (eg, “Did you find the AI-generated explanation helpful?” rated yes or no, and an open comment). These will be reported qualitatively to supplement interpretation, but they are not primary outcomes.

### Sample Size and Power Calculation

We determined the target sample size of 300 patients per trial (150 per arm) through power analysis based on the primary outcome (FAPI score). From previous studies using the FAPI/QQPPI in similar settings, we estimate the mean (SD) FAPI score in usual care to be approximately 3.5 (SD 1.0) on the 5-point scale. We consider an increase of 0.3-0.5 in the mean score (with 0.5 being a medium effect, roughly half an SD) to be a clinically important improvement in communication quality. To detect a difference of 0.5 with 80% power at a 2-sided α of 0.05 using a 2-sample *t* test (2-tailed), approximately 63 patients per group are required. To be conservative and account for potential dropout or non-evaluable responses (eg, incomplete questionnaires), we inflated the sample. Our goal of 300 participants per trial (150 per arm) provides >90% power to detect an effect size of *d*=0.5 and approximately 80% power to detect a smaller effect of *d*≈0.4. This sample also provides ample power (>80%) for detecting differences in secondary outcomes, such as a 10-point difference in the 0-100 scaled DDPRQ-10 or approximately a 1-point difference in the understanding rating. We do not plan an interim efficacy analysis, but an internal check of assumptions, such as variance, will be conducted after approximately 50% enrollment to ensure the sample size remains adequate. Given the consecutive recruitment strategy, we anticipate reaching 300 patients in each trial within the planned 6-month recruitment window, as the neurosurgery service sees dozens of new patients weekly and discharges dozens monthly, with a good proportion expected to consent.

### Data Collection and Management

Data are entered into a pseudonymized, access-controlled database on secure hospital servers with routine quality checks (double-entry for a 10% sample and discrepancy resolution from source forms). Each participant is assigned a study ID; no names or identifying information are entered in the analytic dataset. The patient questionnaires (FAPI, HCCQ, and self-rated understanding) are filled by the patient in a private area after their visit, with research staff available to assist if needed (for example, reading questions aloud if the patient has minor reading difficulties; severe impairment was an exclusion criterion). The physician questionnaire (DDPRQ-10) is filled separately by the physician immediately after the visit, without seeing the patient’s responses. Consultation time is recorded in minutes by staff. Any technical issues (eg, the AI summary not being ready in time) are documented, and those patients are analyzed in their assigned group according to the intention-to-treat principle.

Data management will follow Good Clinical Practice guidelines. Regular data quality checks will be performed, and any discrepancies in entry will be corrected by checking against the source paper. The analysis will primarily be intention-to-treat, including all randomized patients in the groups to which they were assigned. If any patient does not receive the intervention (eg, technical failure to generate the summary) or does not complete outcomes, this will be noted; missing data will be handled through multiple imputation if less than 5% are missing, or through sensitivity analyses excluding those cases.

### Statistical Analysis Plan

We will first describe the sample using summary statistics (means or medians for continuous variables, frequencies for categorical variables). The two trial populations (outpatient vs inpatient) will be described separately. Baseline characteristics will be compared between arms to ensure that randomization achieved balance; any significant differences will be noted and adjusted for in secondary analyses if needed.

For the primary outcome (FAPI score), we will compare the intervention and control group means using an independent-samples *t* test (or nonparametric Wilcoxon rank-sum test if the distribution is highly skewed, although previous studies suggest near-normal distribution). We will report the mean difference with 95% CI. The primary analysis will be carried out separately for each trial. However, since the interventions and outcomes are identical, we may also perform a pooled analysis across both trials to increase power, including an indicator for trial to account for any systematic differences in context. Our hypothesis test is 2-sided with α=0.05. We anticipate a positive difference (intervention > control).

### Secondary Outcomes Analyses

HCCQ scores will be compared similarly using a *t* test. Understanding ratings, which are on a scale from 1 to 5, will be compared using a Mann-Whitney *U* test for ordinal data or by dichotomizing responses as “understood well” for ratings of 4 or 5 and “not well” for ratings of 1-3, followed by a chi-square test. DDPRQ-10 scores, which yield a numeric difficulty score, will be compared by *t* test; additionally, we may compare the proportion of encounters meeting a “difficult” threshold (there is no absolute cutoff in literature, but >30 on the original 0-100 scale is sometimes used). Consultation time (in minutes) is likely to be skewed; we will compare medians with a Wilcoxon test, and also compare proportion of consultations exceeding a certain length (eg, >15 minutes) using a chi-square test.

We will consider *P*<.05 as significant for the primary outcome. For secondary outcomes, *P* values <.01 or <.05 will be interpreted with caution due to multiple comparisons. We are not formally adjusting for multiplicity, given that outcomes are correlated; however, the pattern of results will be considered collectively. Effect sizes will be presented (Cohen *d* for continuous outcomes, odds ratios for categorical comparisons) to gauge practical significance.

### Subgroup and Exploratory Analyses

We plan to explore whether the effect of the intervention differs by subgroups such as age, with older patients potentially benefitting more or less, education level, with those having lower education or health literacy possibly gaining more, and by doctor. To do this, we may run an ANOVA or regression with interaction terms. For example, we will use a linear regression with FAPI score as the outcome, the intervention as predictor, and an interaction term between intervention and health literacy level, if available from a brief screening question, to examine whether the effect varies. These analyses are exploratory and hypothesis-generating.

We will also analyze the results of the two trials side by side to see whether context, specifically outpatient versus inpatient, influences outcomes. If the direction and magnitude of effect are similar across trials, this strengthens generalizability. If one trial shows a different outcome, for instance if in one context the summary is more beneficial, that will be an interesting finding to explore qualitatively.

All analyses will be performed using SPSS (version 28; IBM Corp) and R software (R Foundation for Statistical Computing). Data analysis will commence after all follow-up is complete. No interim analysis for early stopping is planned, as the intervention is low-risk.

## Results

As of May 2025, the trials are in the prerecruitment phase. All regulatory approvals and registrations are in place. Recruitment will commence on May 26, 2025, and is expected to continue until February 2026 or until the target sample sizes are reached. Statistical analysis plans have been finalized prior to trial initiation. Data analysis is expected to begin promptly after completion of data collection. Results are anticipated to be published in summer 2026. Neither study received external funding.

To date, no participants have been enrolled and no interim data are available; therefore, we cannot report any baseline characteristics or outcomes. Enrollment rates will be monitored closely. Based on clinic volumes, we anticipate enrolling approximately 50 outpatients and 25 to 30 inpatients per month, which should allow reaching 300 in each trial within 6 months. If necessary, recruitment may be extended or involve additional centers, but at this time the plan is to keep it single-center and within the 6-month window.

Each trial’s progress and any protocol deviations will be documented. An independent data safety officer will periodically review recruitment and any AEs. We do not expect any physical AEs from this type of intervention. Potential adverse outcomes could include a patient being very upset by understanding their diagnosis; ethically, such reactions are part of informed care. Physicians will manage these situations with appropriate counseling. No such cases have occurred to date, as the trials have not yet started.

We will publish the trial results in a peer-reviewed journal upon completion and also plan to share findings with stakeholders, including patient advocacy groups and the hospital administration for potential implementation. The trial registration details, DRKS00036810 for AI-INFOCARE and DRKS00036814 for AI-MEDTALK, are publicly accessible, and we will update those entries with a results summary after study completion as required by guidelines.

In summary, at this protocol publication stage, we confirm that everything is ready for execution of the trials. The key upcoming milestone will be the enrollment of the first patient, anticipated in late May 2025. We will treat the first few cases as a pilot to ensure logistics, such as timely generation of the AI summary while the patient is waiting, function smoothly, then proceed to full recruitment.

We will present the final analyzed results, including comparisons between the intervention and control group for all outcomes, after the trial is completed. These results will allow us to evaluate our hypotheses on whether AI-driven simplification of medical documents can measurably improve the doctor–patient communication experience.

In addition to peer-reviewed publication, results, whether positive or negative, will be posted on the Deutsches Register Klinischer Studien registry, summarized in patient-readable German on the hospital website, and shared with patient advocacy groups and professional societies. Appendices including standard operating procedures and fidelity metrics, will be made available where feasible.

## Discussion

### Overview

Effective communication is fundamental to patient-centered care, yet achieving it remains challenging in practice. Our protocol describes two novel RCTs addressing a simple but prevalent problem: patients often do not fully understand the written medical information that accompanies their care, which can hinder meaningful dialogue with their doctors. By leveraging AI to translate “doctor language” into “patient language,” we aim to enhance understanding and thereby improve various facets of communication.

The Discussion section examines the rationale for and potential implications of our intervention in light of existing literature, as well as the strengths and limitations of this study design.

### Interpretation of the Intervention’s Potential Impact

If our hypothesis is correct, patients who receive an easy-to-read summary of their medical documents will feel more informed and engaged during consultations. Previous research strongly suggests that when patients comprehend their health information, several positive downstream effects occur. They are more likely to ask questions and participate in decision-making, which is a core aspect of patient-centered communication [2]. They may also experience reduced anxiety because uncertainty is diminished, as an informed patient is less likely to fear the unknown.

A classic review by Stewart [1] found that effective physician–patient communication positively influences outcomes ranging from emotional health to symptom resolution. Our intervention specifically targets the information exchange component of communication [2], aiming to make it more bidirectional and understandable. According to the model of Ong et al [2], better exchange of information, alongside a good interpersonal relationship, should improve patient satisfaction, recall, and adherence. We are measuring satisfaction with communication through FAPI and autonomy support through HCCQ, both of which we expect to improve. Autonomy support, in particular, could be enhanced because patients with preobtained knowledge might feel more control over the conversation and subsequent decisions [11]. This aligns with self-determination theory, which emphasizes that supporting a patient’s autonomy, for instance by providing choices or acknowledging their perspective, leads to better motivation and adherence to treatment plans. In our context, giving the patient information in advance is a way of respecting their role in the decision process, effectively saying, “we want you to understand and be an active partner.” We anticipate this will be reflected in higher HCCQ scores in the intervention group, indicating that patients feel their doctors were more autonomy-supportive. Notably, even without specific training for doctors, an intervention that educates patients can indirectly change the communication dynamic, as doctors might notice the patient is well-prepared and accordingly have a more nuanced discussion rather than spending most of the time explaining basics.

For physicians, one might worry that more informed patients could be more challenging, reflecting the stereotype of the patient arriving with a stack of internet printouts. However, research often contradicts this worry: physicians generally appreciate when patients have some baseline knowledge, provided it is accurate, because it can save time and lead to more focused questions [7]. Difficult physician–patient interactions often stem from misalignment of expectations or repeated explanation of basic issues [13]. We posit that our summaries will alleviate those factors, which is why we measure DDPRQ-10 to determine whether doctors find these conversations less frustrating or difficult. A reduction in “difficult” encounters is beneficial not only for patient care but also for physician well-being. Studies show that perceived difficult encounters correlate with physician burnout and suboptimal care [12]. If a simple intervention can reduce that strain, it represents a win–win. Of course, we must be cautious: it is possible that some doctors might feel the AI summary could undermine their role as information-giver or they might worry about inaccuracies. We have attempted to mitigate these concerns by involving physicians in validating the summaries and making clear that the tool is an aid, not a replacement for their explanations.

### Comparison With Related Work

Our approach draws upon previous work in health literacy and patient education interventions. There have been efforts to rewrite medical documents in simpler language manually. For instance, Smolle et al [7] simplified discharge instructions, and found improved patient comprehension [7,8]. However, manual simplification is labor-intensive and not scalable in routine practice. The innovative aspect here is using AI (LLMs) to do this at scale and on-demand. Generative AI has only recently become capable enough to handle such tasks with reasonable accuracy. Zaretsky et al [8] provided evidence that an LLM (GPT-4 via Azure) can reliably simplify discharge summaries while maintaining content integrity. They did highlight safety concerns—in 18 of 100 reviews, physicians noted some missing or inaccurate information in AI outputs. This underscores the importance of having a human in the loop, as we have in our protocol. Our results will contribute real-world data on whether an AI-assisted workflow can be integrated feasibly without error-induced AEs.

Another related area is the use of patient question prompt lists or decision aids to improve communication. Those interventions provide patients with structured information and questions to consider, often leading to improved question-asking and involvement. Our AI summary could function similarly to a tailored information sheet; it might even prompt patients to ask about things they read. We will observe qualitatively if patients in the intervention ask more questions. If so, that could be an added benefit—engaged patients often lead to more accurate diagnoses and more concordant treatment plans [2].

In terms of health outcomes, our trials are not measuring long-term outcomes like adherence or health status. But literature ties improved communication to those outcomes. For example, a meta-analysis by Jahan and Siddiqui [14] found that good physician communication is associated with higher adherence to treatment (odds of adherence were 2.16 times higher when patients rated their physician’s communication as excellent). While beyond our scope, it is conceivable that if patients understand their condition and instructions better (through the summary and ensuing discussion), they will be more likely to follow medication and follow-up plans. We plan future research to track such outcomes.

### Public Health and System Implications

Health literacy has been called “the currency of health” in modern health care. Germany, like many countries, has recognized the need to improve health literacy at a population level [1]. Our intervention is one piece of that puzzle—a tool that health care providers can use to ensure each patient, regardless of background, receives information in an understandable form. If our trials show positive results, it would support wider implementation of AI-generated patient-friendly documents across hospitals and clinics. This could be particularly impactful in systems with high patient throughput and limited time, such as busy outpatient practices. By making written communications clearer, the onus on the doctor to cover everything orally in limited time might lessen, as patients come in with some pre-understanding.

From a system perspective, one might worry that providing these summaries is extra work or cost. However, thanks to AI automation, once set up, generating a summary takes only seconds and could be triggered as part of the electronic health record workflow. The physician’s time to review the summary for accuracy is a few minutes—likely offset by time saved in repetitive explanations. And the cost of using the AI (computing cost) is minor per document—perhaps a few cents to a couple of euros depending on length and model pricing. There is also no external funding for this study, meaning this concept is being developed without pharmaceutical or large grant backing—a testament that it is a practical solution emerging from clinical need and tech opportunity, rather than a commercial product push. If effective, we imagine no difficulty in convincing hospital administrations to adopt it, given the relatively low cost and alignment with quality improvement goals (some countries might even incorporate it into guidelines for discharge processes to improve patient comprehension).

Another important aspect is generalizability. While this study is in a neurosurgery context with German-language documents, the principle is applicable to any specialty and any language where a robust translation model exists. Chronic illness management, for example, could benefit—patients with diabetes or cancer often receive complex written instructions, and simplifying those could empower them in self-care. Primary care referrals or interspecialist letters could also be translated so patients fully know what is being communicated about them. On a global level, organizations like the Organisation for Economic Co-operation and Development emphasize patient-centered care and health literacy; our approach directly operationalizes some of the Organisation for Economic Co-operation and Development’s recommendations for using digital tools to improve communication and patient engagement. It exemplifies how digital health innovation can tackle the enduring issue of jargon-filled health care.

### Potential Challenges and Limitations

It is important to consider potential limitations and how they will be addressed.

#### Accuracy of AI Output

While we have a verification step, there is a risk that an AI-generated summary omits a nuance or contains a subtle error. If not caught, this could mislead a patient. For instance, if a summary accidentally downplayed a serious finding, the patient might not realize its importance. We mitigate this with human review and by instructing doctors to double-check understanding. In our future results, we will audit how often corrections were needed. If too many corrections are needed, that could limit scalability. However, given the continuous improvement of LLMs, we expect accuracy to only improve over time. Our current safety plan appears robust for trial purposes.

#### Effects on Consultation Dynamics

We assume the effect is positive, but it is possible some doctors or patients might react unexpectedly. A doctor might feel that the patient’s preknowledge encroaches on their explanatory routine, or a patient might feel overloaded if they read a lot before the talk. It is a delicate balance—one could liken it to giving patients laboratory results directly before talking to the doctor; some studies show patients prefer knowing results beforehand, while others caution that it can raise anxiety. Our trials will provide insight on this. Through the FAPI and qualitative feedback, we will see if any negative sentiments arise, for example, a patient could say “reading it alone made me worried.” We believe the net effect is beneficial as long as the subsequent conversation addresses any anxiety the information caused. Indeed, knowing what to be worried about can lead to addressing it head-on with the physician, which is ultimately good.

#### Generalizability to Lower Literacy Populations

This study requires German proficiency. Those with very low literacy might still struggle, even with a simplified summary, although our summaries aim for fairly low reading level. If someone cannot read at all, our intervention does not help, and they would be excluded. In practice, family members could read to them, but we did not formalize that. Thus, the intervention mostly aids those who can read basic German but not medical German, which is a large group. Future solutions could include audio or video explanations, with AI generating spoken or visual content. This is beyond our current scope but is worth exploring.

#### Two Trials Versus One

We chose to run two separate trials for two scenarios. This is both a strength, covering more ground, and a complexity. It is possible the effect size differs between them. For instance, outpatients might benefit more because they have time to process information before making a decision, whereas inpatients at discharge may be still recovering and less able to absorb written information. Alternatively, inpatients might have a more immediate need to understand postoperative care. If one trial shows significant improvement and the other does not, interpretation will need to consider contextual differences such as pain, stress, and urgency. However, each trial on its own is powered and important. We will report them separately, as well as discuss combined lessons. We consider them as complementary pieces of evidence. Running two complementary RCTs allows us to capture distinct communication inflection points: preparing for treatment decisions in outpatients (AI-INFOCARE) and understanding postsurgical instructions at discharge (AI-MEDTALK). These contexts pose different informational and emotional demands. Analyzing both separately and in pooled form strengthens external validity and clarifies whether effects generalize across settings or are context-dependent.

#### No Blinding and Potential Bias

Because patients know if they got a summary, their responses could be influenced by a sort of placebo or novelty effect; they might rate the communication highly out of gratitude or perception that they got extra care. Similarly, physicians know which patients got summaries and might unconsciously treat them a bit differently, perhaps spending a bit less time explaining basics. These are real possibilities. However, they are part of the intervention’s effect in a pragmatic sense—in real use, everyone would know the summary is given, and that itself could change behaviors; doctors in future might rely on these tools and adjust their approach accordingly. We opted for a pragmatic trial perspective: measure the outcome as it occurs with knowledge of the intervention. To gauge if bias is substantial, we will compare objective measure of time. If patients report much higher understanding but in reality asked many questions or time increased, we might consider that a bias in perception. We will triangulate data to judge this.

#### Single-Center, Single-Department Setting

Our sample comprises all neurosurgery patients, which may limit generalizability. Communication needs in neurosurgery might differ from, for example, internal medicine. Nonetheless, many issues, such as complex terms and short visits are universal. We deliberately included heterogeneity within neurosurgery, including spine issues, brain tumors, and other conditions across a broad adult age range, to obtain a diverse sample. Still, future multicenter trials in other fields would be valuable. For instance, applying this approach in primary care or oncology could be next steps. We anticipate our results will encourage such studies or implementations. Thus, while this study provides a proof-of-concept in neurosurgery, results may not directly extrapolate to other specialties. Future multicenter studies across diverse fields, such as oncology or primary care, are needed before broad implementation.

#### Risk of Workflow Disruption

Another important limitation is the risk of workflow disruption. Physicians may experience additional cognitive load from reviewing AI outputs, and patients may experience heightened anxiety if sensitive diagnoses are clearly stated. Furthermore, AI errors, even if rare, could undermine trust. These risks are integral to the evaluation and will be systematically monitored through the fidelity audit and AE reporting.

### Expected Outcomes and Future Directions

We anticipate that the Discussion section in the results paper will highlight improvements in the intervention group across most measures. If, for example, we find that patients with AI summaries have significantly higher FAPI scores, indicating a better perceived interaction, and that their understanding ratings are substantially better, it will provide direct evidence that understanding written information translates to feeling of better communication. We also expect to see a small improvement in satisfaction, which the FAPI captures, and autonomy support, as measured by the HCCQ, possibly moderately higher. We are very curious about the consultation length outcome. A noninferiority result, showing no significant increase in time, would be encouraging because it means such summaries do not slow down clinics. Even if time increases by a minute or two, if outcomes are improved, many would argue it is worth it, as that time might be offset by fewer phone calls later.

One intriguing possibility is that improved understanding could reduce decisional conflict. Although we are not formally measuring decisional conflict in this protocol, some FAPI items indirectly relate to whether patients felt involved in decisions. If analysis of those items, such as “The doctor and I made treatment decisions together,” shows higher agreement in the intervention group, that implies better SDM. This aligns with findings that information provision is a prerequisite for SDM.

After these trials, we foresee a need to evaluate longer-term outcomes. For example, do these better communication experiences lead to improved adherence to discharge instructions or follow-up attendance? Are there fewer calls to the clinic with questions, or less need for repeated explanations? We have heard anecdotal reports that patients often call days after discharge with questions indicating they did not grasp the discharge instructions. If our intervention works, those calls might be reduced; we may try to gather such data as an exploratory measure during the trial. Additionally, health outcomes such as blood pressure control, pain management, or recovery might indirectly benefit when patients truly understand their care plan. It would be worth examining specific cases, such as medication adherence after discharge; this could be a follow-up study. Some neurosurgery patients receive new medications, such as steroids or anticonvulsants, and comprehension of instructions for those is vital.

Another future direction is cost-effectiveness. Although this study is not an economic evaluation, if it shows improved outcomes, the next question is at what cost, or potentially savings? Given the minimal costs for AI text generation, the main cost is personnel time for review and implementation. If the approach reduces readmissions or unnecessary visits due to confusion, it could even be cost-saving. We will gather some data on postdischarge contacts that may feed into a later cost analysis.

Finally, our research contributes to the broader field of how digital tools can support health communication without replacing the human touch. We emphasize that this AI tool is not taking over the physician’s role; rather, it augments the communication process. This distinction is important in discussions about AI in medicine. By focusing on translation of language, we are using AI in a targeted, low-risk way that potentially yields high impact. The trials’ outcomes will inform not only this specific use case but also how acceptability of AI interventions is perceived by patients. If patient satisfaction remains high, or even improves, with an AI-assisted process, that bodes well for patient acceptance of other AI-supported health care tasks.

### Conclusion

In conclusion, the AI-INFOCARE and AI-MEDTALK trials will shed light on a promising strategy to improve doctor–patient communication by addressing comprehension barriers. These studies are timely, given the dual trends of rising health information complexity and the emergence of AI solutions. A strength of our approach is that it aligns with fundamental ethical imperatives in medicine: respect for persons by informing them, beneficence by potentially improving outcomes, and justice by making information accessible to those who might otherwise not understand it. It operationalizes the recommendation that health information be made understandable as a means to improve health literacy on an individual level [1].

If successful, we envision that providing AI-generated layperson-friendly summaries could become a standard part of the workflow for many clinical encounters—patients routinely receiving two versions of every important document: one for clinicians and one for themselves. Our research will provide evidence on whether this indeed improves the patient experience and what pitfalls to watch for, such as ensuring accuracy of AI output. It may also encourage health systems to invest in patient education through technology, which historically sometimes lags behind clinician-facing technology investments. As health care increasingly adopts digital health records and patient portals, integrating an AI summarization feature is a logical next step; these trials will clarify the value of doing so.

The discussion around doctor–patient communication has often highlighted training doctors to speak more clearly and checking patient understanding using methods such as teach-back. Those remain crucial, but our intervention tackles the problem from another angle, modifying the written side of communication. In a sense, it relieves some burden from the spoken conversation by preinforming the patient. We believe these approaches—improving written information and improving oral communication skills—are complementary and both should be pursued to maximize understanding.

Ultimately, the goal is captured well by a simple idea: when patients truly understand their health situation, they are better equipped to care for themselves and engage with their providers. Our trials aim to provide rigorous evidence that a cutting-edge AI tool can help achieve that understanding in everyday clinical practice. The results, once available, will contribute to the evolving narrative of how technology can humanize medicine by fostering knowledge, trust, and partnership between patients and doctors, rather than detracting from it.

Our findings will add to a growing body of research on digital health literacy and AI-assisted patient communication. Recent JMIR publications have highlighted both the opportunities and risks of LLM-mediated patient materials, showing that readability can be improved substantially, but accuracy and safety remain critical [15,16]. Evidence from digital health literacy interventions demonstrates measurable benefits on comprehension and engagement [17]. Work on optimizing plain-language summaries further supports the importance of tailoring complexity to patient needs [18]. By embedding our protocol within this emerging literature, we extend previous findings into a pragmatic, RCT framework and provide real-world evidence of feasibility.

While this protocol focuses on German-speaking patients in a single neurosurgical center, the approach could be extended to other populations. Potential adaptations include tailoring summaries for patients with low literacy, generating multilingual versions, or providing information in alternative formats such as audio or video. These directions may broaden accessibility and scalability beyond the present setting and will be considered in subsequent studies.

## Appendix

Multimedia Appendix 1 Correction log template.

## Abbreviations

AE: adverse event
AI: artificial intelligence
DDPRQ-10: Difficult Doctor–Patient Relationship Questionnaire, 10-item
FAPI: Fragebogen zur Arzt-Patient-Interaktion
HCCQ: Health Care Climate Questionnaire
LLM: large language model
QQPPI: Questionnaire on the Quality of Physician–Patient Interaction
RCT: randomized controlled trial
SDM: shared decision-making
SPIRIT: Standard Protocol Items: Recommendations for Interventional Trials
