# Supplementary material for: Layperson-Friendly AI Translation of Medical Documents to Improve Doctor–Patient Communication: Protocols for the AI-INFOCARE and AI-MEDTALK Randomized Controlled Trials
Source: JMIR Res Protoc. 2025 Nov 21;14:e77204. doi: 10.2196/77204 (PMC12680933; doi:10.2196/77204)
Supplement: Multimedia Appendix 1 [file resprot_v14i1e77204_app1.pdf]

## Appendix A4. Planned Correction Log Template for AI-Generated Summaries

**Purpose:**

This appendix presents the predefined template that will be used to document and categorize issues detected during human validation of AI-generated lay summaries. As the trials have not yet commenced, no entries are currently available. The template ensures transparency and reproducibility of the planned monitoring process.

**Correction Log Template**

| Patient/Case ID | Date | Reviewer ID | Prompt Version | Error Category* | Description of Issue | Correction Applied | Verified by (2nd Reviewer if applicable) |
|---|---|---|---|---|---|---|---|
| Example 001 | 2025-06-01 | R01 | V1.0 | Omission | Missing follow-up appointment details | Added based on source document | R02 |
| Example 002 | 2025-05-02 | R03 | V1.0 | Risk attenuation | “Urgent MRI required” simplified to “MRI recommended” | Restored urgency wording | – |

**Error Categories:**

- Omission – relevant information missing
- Mislabeling – incorrect medical term or patient detail
- Risk attenuation – urgency or severity understated
- Addition – content not present in original document
- Contradiction – conflict between summary and source
- Formatting/Clarity – structural or readability issues only

**Planned Audit:**

- Quarterly audits will calculate the proportion of summaries requiring correction.
- Error rates will be reported by category and by prompt version.
- Interrater agreement (Cohen’s κ / ICC) will be computed for the 10% of summaries with double review.
